# Supplementary material for: ATP-Dependent Clp Protease Subunit C1, HvClpC1, Is a Strong Candidate Gene for Barley Variegation Mutant luteostrians as Revealed by Genetic Mapping and Genomic Re-sequencing
Source: Front Plant Sci. 2021 Apr 16;12:664085. doi: 10.3389/fpls.2021.664085 (PMC8086601; doi:10.3389/fpls.2021.664085)
Supplement: Supplementary Figure 1 — Genetic mapping of the HvLST gene. [file Data_Sheet_1.docx]

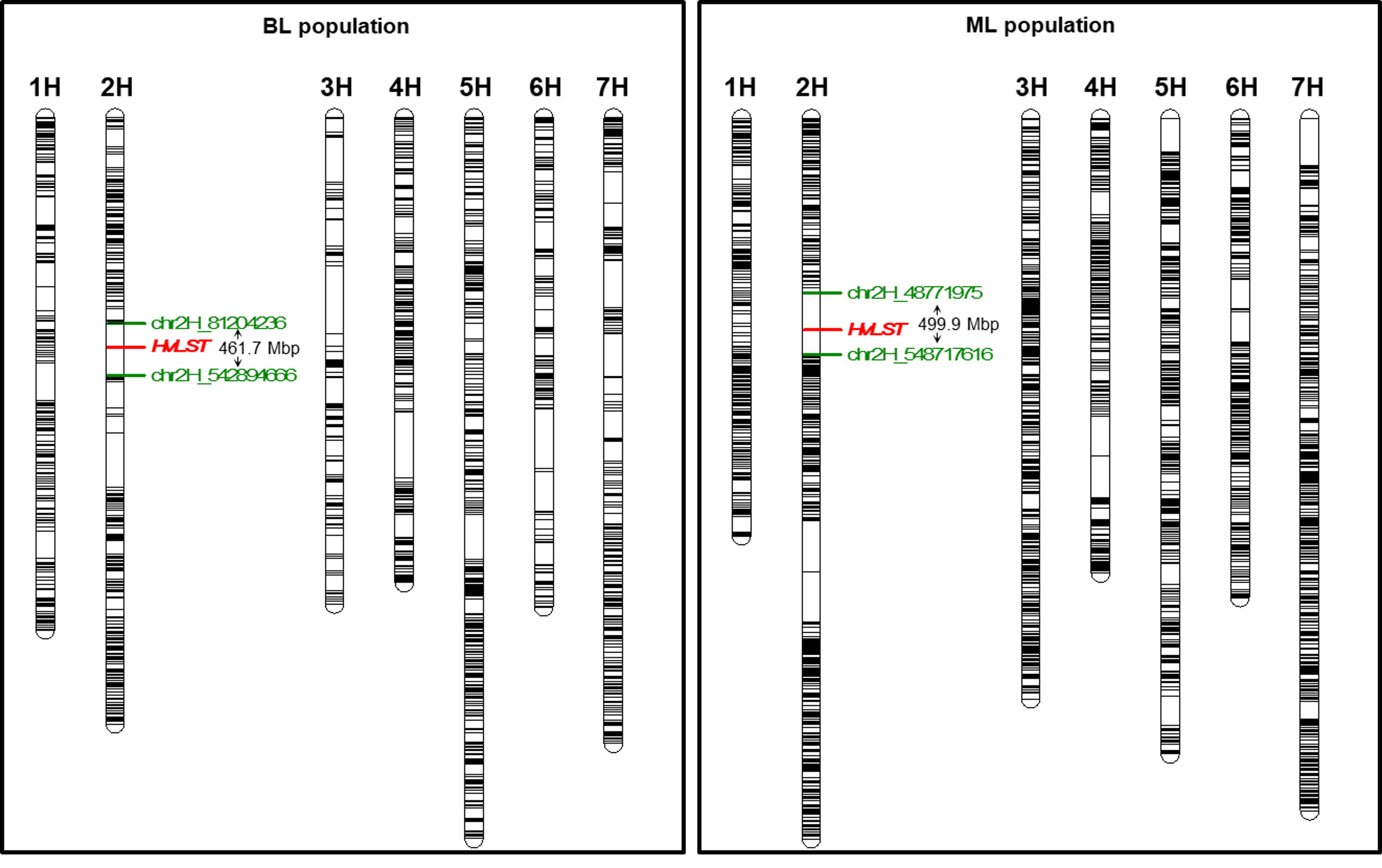


**Supplemental Figure 1. Genetic mapping of the *HvLST* gene.** Genetic mapping of the gene *HvLST* in the BL and ML mapping populations with 124 and 146 F2 genotypes, respectively, based on a genotyping-by-sequencing assay. In both populations, the *HvLST* gene (indicated in red) allocated to chromosome 2H in the close to the genetic centromere. The respective closest flanking markers are indicated in green. The numerical value in the marker designation indicates the physical coordinate of the mapped SNP position on the reference genome of barley (Monat et al., 2019). The flanking markers therefore delineate a physical distance of 461.7 Mbp and 499.9 Mbp in BL and ML populations, respectively.

| **Supplemental Table 1. Summary of SNP markers derived from genotyping-by-sequencing** | | | | |
| --- | --- | --- | --- | --- |
| Chromosome | **BL Population (124 genotypes)** | | **ML Population (146 genotypes)** | |
|  | # of markers | # of mapped markers | # of markers | # of mapped markers |
| 1H | 434 | 300 (69.1%) | 556 | 393 (70.7%) |
| 2H | 656 | 423 (64.5%) | 856 | 608 (71.0%) |
| 3H | 243 | 166 (68.3%) | 812 | 576 (70.9%) |
| 4H | 450 | 304 (67.6%) | 460 | 335 (72.8%) |
| 5H | 707 | 472 (66.8%) | 707 | 514 (72.7%) |
| 6H | 308 | 204 (66.2%) | 570 | 414 (72.6%) |
| 7H | 571 | 369 (64.6%) | 893 | 629 (70.4%) |
| Total | 3369 | 2238 (66.4%) | 4854 | 3469 (71.5%) |

| **Supplemental Table 2. Graphical genotype of selected F2 recombinants** | | | | | | | | | | | | | | | | | |
| --- | --- | --- | --- | --- | --- | --- | --- | --- | --- | --- | --- | --- | --- | --- | --- | --- | --- |
| **BL F2 Population** | | | | | | | | | | | | | | | | | |
| F2 plant ID | chr2H_178489464 | chr2H_210003353 | chr2H_229488641 | chr2H_350172206 | chr2H_380410256 | chr2H_400397603 | chr2H_420602111 | chr2H_426041295 | chr2H_431057673 | *HvLST* | chr2H_436006836 | chr2H_437054630 | chr2H_440013544 | chr2H_450060325 | chr2H_455041513 | chr2H_458001177 | chr2H_461744325 |
| MZ38-2_3_19 | H | H | H | H | H | H | H | H | H | H | H | H | H | H | H | - | A |
| MZ38-2_3_70 | A | A | A | A | H | H | H | H | H | H | H | H | H | H | H | H | H |
| MZ38-2_3_97 | H | H | H | H | H | H | H | H | H | A | A | A | A | A | A | A | A |
| MZ38-2_5_12 | H | A | A | A | A | A | A | A | A | A | A | A | A | A | A | A | A |
| MZ38-2_5_17 | H | H | H | H | H | H | H | H | H | - | H | H | H | H | H | - | A |
| MZ38-2_5_21 | A | A | A | A | A | A | A | A | H | H | H | H | H | H | H | H | H |
| MZ38-2_5_41 | B | H | H | H | H | H | H | H | H | H | H | H | H | H | H | H | H |
| MZ38-2_5_66 | H | A | A | A | A | A | A | A | A | A | A | A | A | A | A | A | A |
| MZ38-2_5_97 | A | A | A | A | A | A | A | A | A | A | A | A | A | A | A | H | H |
| MZ38-2_5_121 | B | H | H | H | H | H | H | H | H | H | H | H | H | H | H | H | H |
| MZ38-2_5_127 | H | H | H | H | H | H | H | H | H | H | H | H | H | H | H | A | A |
| MZ38-2_5_136 | A | A | A | A | A | A | A | A | A | A | A | A | A | A | A | A | H |
| MZ38-2_5_143 | A | A | A | A | A | A | A | A | A | H | H | H | H | H | H | H | H |
| **ML F2 Population** | | | | | | | | | | | | | | | | | |
| F2 plant ID | chr2H_200083049 | chr2H_210003353 | chr2H_229488641 | chr2H_350172206 | chr2H_380410256 | chr2H_400397603 | chr2H_420602111 | chr2H_426041295 | chr2H_431057673 | *HvLST* | chr2H_436006836 | chr2H_437054630 | chr2H_440013544 | chr2H_450060325 | chr2H_455041513 | chr2H_458001177 | chr2H_463274491 |
| MZ38-1_4_15 | H | A | A | A | A | A | A | A | A | A | A | A | A | A | A | A | A |
| MZ38-3_2_56 | H | A | A | A | A | A | A | A | A | A | A | A | A | A | A | A | A |
| MZ38-3_2_57 | H | H | H | H | H | H | H | H | H | H | H | H | H | H | H | H | B |
| MZ38-3_2_58 | B | H | H | H | H | H | H | H | H | H | H | H | H | H | H | H | H |
| MZ38-24_2_96 | H | A | A | A | A | A | A | A | A | A | A | A | A | A | A | A | A |
| A- wild type; H- Heterozygote; B- Homozygous mutant; missing data is represented by a dash symbol '-'. | | | | | | | | | | | | | | | | | |
| The flanking markers are indicated in red and the *HvLST* locus is indicated in green. | | | | | | | | | | | | | | | | | |

| **Supplemental Table 3. Summary of candidate genes for *HvAST* and *HvLST*** | | | | | | |
| --- | --- | --- | --- | --- | --- | --- |
| **Gene ID^A^** | **Confidence** | **Original^B^** | **SNP** | **Effect** | **SNP position^C^** | **Location** |
| **Candidate genes for *HvAST*** | | | | | | |
| ***HORVU.MOREX.r2.7HG0603920.1*** | HC | **ATAC** | Deletion | Immature stop codon | 1123-1126 | Exon 1 |
| *HORVU.MOREX.r2.7HG0603970.1* | LC | CA**C** | C/G | H731Q | 2193 | Exon 2 |
| *HORVU.MOREX.r2.7HG0604040.1* | HC | G**A**C | A/G | D67G | 200 | Exon 1 |
| *HORVU.MOREX.r2.7HG0604110.1* | HC | **A** | **A**CATAC | n.a | n.a | Intron 9 |
| **Candidate genes for *HvLST*** | | | | | | |
| *HORVU.MOREX.r2.2HG0133160.1* | HC | **C**AC | C/T | H310Y | 928 | Exon 3 |
| *HORVU.MOREX.r2.2HG0133350.1* | HC | **G**CG | G/T | A306S | 916 | Exon 1 |
| *HORVU.MOREX.r2.2HG0133900.1* | LC | **G**TT | G/A | V189I | 565 | Exon 1 |
| *HORVU.MOREX.r2.2HG0134330.1* | HC | C**C**A | C/T | P150T | 449 | Exon 6 |
|  |  | A**A**T | A/G | N151S | 452 | Exon 6 |
|  |  | AA**G** | G/T | K156N | 468 | Exon 6 |
|  |  | G**A**A | A/T | E419V | 1256 | Exon 8 |
|  |  | **C**CA | C/G | P454A | 1360 | Exon 8 |
|  |  | A**C**C | C/T | T462I | 1385 | Exon 8 |
|  |  | C**G**C | G/A | R500H | 1499 | Exon 9 |
|  |  | G**G**A | G/T | G583V | 1748 | Exon 9 |
|  |  | C**A**G | A/G | Q584R | 1751 | Exon 9 |
|  |  | **A**AA | A/C | K589Q | 1765 | Exon 9 |
|  |  | GA**G** | G/C | E592D | 1776 | Exon 9 |
|  |  | **G**AA | G/C | E593Q | 1777 | Exon 9 |
| *HORVU.MOREX.r2.2HG0134390.1* | HC | G**G**T | G/A | G47D | 140 | Exon 2 |
| *HORVU.MOREX.r2.2HG0134800.1* | HC | T**A**T | A/T | Y14F | 41 | Exon 1 |
| *HORVU.MOREX.r2.2HG0135080.1* | HC | **A**CC | A/G | T198A | 1492 | Exon 3 |
| *HORVU.MOREX.r2.2HG0135330.1* | HC | **G**AT | G/A | D67N | 199 | Exon 1 |
| ***HORVU.MOREX.r2.2HG0135340.1*** | HC | G**G**T | G/A | G693D | 2078 | Exon 9 |
| *HORVU.MOREX.r2.2HG0135360.1* | HC | C**G**T | G/A | R121H | 362 | Exon 1 |
| *HORVU.MOREX.r2.2HG0135690.1* | HC | G**C**G | C/T | A56V | 167 | Exon 1 |
| ^A_^The gene ID in bold indicates the *HvAST* locus and potential *HvLST* locus. | | | | | | |
| ^B_^The SNP position is marked in bold. | | | | | | |
| ^C_^Coordinates based on coding sequence of cv. Morex. The Adenine of start codon is counted as position +1. | | | | | | |

| **Supplemental Table 4. Primers used in this study** | | | |
| --- | --- | --- | --- |
| KASP marker ID | Allele 1 (x-axis Primer) | Allele 2 (y-axis Primer) | Common Reverse Primer |
| chr2H_200083049 | GAAGGTGACCAAGTTCATGCTGCCATTTCAGCCATCGTTCCAC | GAAGGTCGGAGTCAACGGATTGGCCATTTCAGCCATCGTTCCAT | GAGAATGTTCCGTTTGGCAGTGCAT |
| chr2H_229499954 | GAAGGTGACCAAGTTCATGCTAGCCTAAGCCGGTAGCAGCAA | GAAGGTCGGAGTCAACGGATTGCCTAAGCCGGTAGCAGCAG | TAACCAGGGGCACTCGGAGCA |
| chr2H_178489464 | GAAGGTGACCAAGTTCATGCTAGCATCCCTCGCCCCAGCT | GAAGGTCGGAGTCAACGGATTAGCATCCCTCGCCCCAGCA | CTGCTGCTGCGGCAGGGGAT |
| chr2H_461744325 | GAAGGTGACCAAGTTCATGCTGACCCTAAATTGGAAAGTGCC | GAAGGTCGGAGTCAACGGATTACCTGACCCTAAATTGGAAAGTGCT | GTGTTAAAAAACGTCTTACATTATGGGACA |
| chr2H_205010822 | GAAGGTGACCAAGTTCATGCTAAGATGATAACACGAAGCTGAAATGAC | GAAGGTCGGAGTCAACGGATTCAAGATGATAACACGAAGCTGAAATGAT | GAAGGAGTGTTTCTTTCAAATCTTGGTCTA |
| chr2H_210003353 | GAAGGTGACCAAGTTCATGCTCTCTATCCGTTTGTTTCGAGGG | GAAGGTCGGAGTCAACGGATTAGTCTCTATCCGTTTGTTTCGAGGA | CTGGTCAGGGGTAACATGGTCTTTT |
| chr2H_215049919 | GAAGGTGACCAAGTTCATGCTGCGATGTGTTACAGGCTGGATTC | GAAGGTCGGAGTCAACGGATTATGCGATGTGTTACAGGCTGGATTT | TGTCATGTGTCGGGACCCCGAT |
| chr2H_220072745 | GAAGGTGACCAAGTTCATGCTATATTAAAACGTGTTAGTTTAGGTCTTGAC | GAAGGTCGGAGTCAACGGATTAAATATTAAAACGTGTTAGTTTAGGTCTTGAT | ATAGCATCTCCGAACTTCCCCCATA |
| chr2H_225027914 | GAAGGTGACCAAGTTCATGCTTTCTTGGATGAGTCTAAGCTACG | GAAGGTCGGAGTCAACGGATTCTTTCTTGGATGAGTCTAAGCTACA | CTTCTTCTTCTCTTCTTGCTTCTTCTCTT |
| chr2H_229488641 | GAAGGTGACCAAGTTCATGCTAGGGACGCACCGCCACC | GAAGGTCGGAGTCAACGGATTAGGGACGCACCGCCACG | CTCCTGCCAGGCACGGCGT |
| chr2H_229506656 | GAAGGTGACCAAGTTCATGCTAAAAGATTCCGACTGGATGAAGATAG | GAAGGTCGGAGTCAACGGATTCAAAAGATTCCGACTGGATGAAGATAA | GGAAATGTAGTCCGAGTGTTTGCGTA |
| chr2H_229754676 | GAAGGTGACCAAGTTCATGCTATGGTAACTGAAGTGAAGCTGTGC | GAAGGTCGGAGTCAACGGATTGATGGTAACTGAAGTGAAGCTGTGT | CATGGGAAATGGTTCCAAGGTGGAT |
| chr2H_300045848 | GAAGGTGACCAAGTTCATGCTTCTTGAGAAGTATTGAATAGAGATGAAAC | GAAGGTCGGAGTCAACGGATTCTTCTTGAGAAGTATTGAATAGAGATGAAAT | CATTTCAATTTCTACGGGTGTTTTGTTCAA |
| chr2H_350172206 | GAAGGTGACCAAGTTCATGCTCCACGCCAGCCACCGCG | GAAGGTCGGAGTCAACGGATTAACCACGCCAGCCACCGCA | GAGCATGCACTTCAACGGAGCCTT |
| chr2H_450060325 | GAAGGTGACCAAGTTCATGCTCATACTCAGTTTGAAATGGAAACTCTGT | GAAGGTCGGAGTCAACGGATTATACTCAGTTTGAAATGGAAACTCTGG | TGGTAATGTGCCTTCCGTTTGCCAA |
| chr2H_380410256 | GAAGGTGACCAAGTTCATGCTAATGATATATTTAAACCCAACAATTCTTTGATC | GAAGGTCGGAGTCAACGGATTAATGATATATTTAAACCCAACAATTCTTTGATT | ATAACTCAATGAAGAAACCGTGGGTTGAA |
| chr2H_400397603 | GAAGGTGACCAAGTTCATGCTCGTAGCACAGACATTGTAGGTTC | GAAGGTCGGAGTCAACGGATTCCGTAGCACAGACATTGTAGGTTT | GGGTTCAAAGTAATCATTGGAGGAAGAAA |
| chr2H_420602111 | GAAGGTGACCAAGTTCATGCTGTATCTTTACATCTCTTTAAAACTCGTTCAAT | GAAGGTCGGAGTCAACGGATTATCTTTACATCTCTTTAAAACTCGTTCAAG | CTGCGTTTACGAATCTTCTTTTGCTGATT |
| chr2H_440013544 | GAAGGTGACCAAGTTCATGCTCAAAGTTAATAGGAAGATTTTCCCTTTATC | GAAGGTCGGAGTCAACGGATTCAAAGTTAATAGGAAGATTTTCCCTTTATG | TCCAATAGCATGTACCAAATTAATGCCTTT |
| chr2H_455041513 | GAAGGTGACCAAGTTCATGCTAGCGATCATTCTACCTGTCATACTAA | GAAGGTCGGAGTCAACGGATTGCGATCATTCTACCTGTCATACTAG | AGTGCCGTAGCTCCGTTCTATGTTT |
| chr2H_426041295 | GAAGGTGACCAAGTTCATGCTCAAAGCGAGATTCATTCGAGATATC | GAAGGTCGGAGTCAACGGATTCTCAAAGCGAGATTCATTCGAGATATT | CTTTTTTGGAACCATGACTAGATTGCCAA |
| chr2H_431057673 | GAAGGTGACCAAGTTCATGCTGGTAACGGCTCGTCCATTCCC | GAAGGTCGGAGTCAACGGATTGGTAACGGCTCGTCCATTCCT | TGTTGAGGGTAAAGGGAGGGAGTTT |
| chr2H_436006836 | GAAGGTGACCAAGTTCATGCTCGGTGATCGAAAGCGTAAGC | GAAGGTCGGAGTCAACGGATTCCTCGGTGATCGAAAGCGTAAGT | AAGGGGCTTTTGTATTCGGCTCCAT |
| chr2H_437054630 | GAAGGTGACCAAGTTCATGCTAATAAAGGTTGCTACCATAAGGGGC | GAAGGTCGGAGTCAACGGATTATAATAAAGGTTGCTACCATAAGGGGA | TATGCCAAATCCCTTAGTGCAAAGGAAT |
| chr2H_458001177 | GAAGGTGACCAAGTTCATGCTCGGCAAAGATAAGCCTACTTTGAATAT | GAAGGTCGGAGTCAACGGATTGGCAAAGATAAGCCTACTTTGAATAC | CGGTACCAAAGTGGCCACGGAT |
| Primer ID | Forward | Reverse | Purpose |
| Hv2HG0135340 | TGGCAGGCTGGGGAATTAAG | TCGATGACACTGCTTCCGAC | SNP validation |
